# Musculoskeletal complaints in patients with Familial Mediterranean Fever

**DOI:** 10.1186/1546-0096-13-S1-P85

**Published:** 2015-09-28

**Authors:** ZB Özçakar, S Şahin-Kunt, S Özdel, F Yalçınkaya

**Affiliations:** 1 Ankara University, Pediatric Rheumatology, Ankara, Turkey

## Question

Familial Mediterranean fever (FMF) is an autosomal recessive disease, characterised by recurrent, self limited attacks of fever with serositis. The aim of our study was to describe the frequency of musculoskeletal complaints in children with FMF and investigate the effect of genotype on these findings.

## Methods

Files of patients who had been seen in our department (during routine follow-up visits) between January 2013 and January 2014 were retrospectively evaluated. Patients with two mutations were divided into 3 groups; M694V/M694V, M694V/Other mutation and patients carrying two mutations other than M694V. Patients with one mutation were divided into 2 groups; M694V and non M694V carriers.

## Results

The study group comprised 317 FMF patients (170 females, 147 males) with a mean age of 12.2 ± 5.7 years. The frequency of musculoskeletal complaints were as follows; arthritis 18%, arthralgia 43%, leg pain 43%, heel pain 36%, myalgia 8%, protracted arthritis 2%, protracted febrile myalgia 2%. Leg pain and heel pain were more frequently detected in patients with homozygous M694V (p<0.05). Among patients with heterozygous mutations; children with M694V mutation had more frequently artralgia, leg pain and heel pain (p<0.05).

## Conclusions

Musculoskeletal problems were common complaints in patients with FMF. Genotype seems to effect the frequency of these problems and M694V mutation is a predisposing factor for musculoskeletal complaints.

